# Intraoperative Hemoadsorption in Heart Transplant Surgery: A 5-Year Experience

**DOI:** 10.3390/jcdd12040119

**Published:** 2025-03-28

**Authors:** Nikola Sliskovic, Gloria Sestan, Savica Gjorgjievska, Davor Baric, Daniel Unic, Josip Varvodic, Marko Kusurin, Dubravka Susnjar, Sarah Singer, Igor Rudez

**Affiliations:** Department of Cardiac and Transplant Surgery, Dubrava University Hospital, 10000 Zagreb, Croatiasarahsingerm@gmail.com (S.S.); rudi@kbd.hr (I.R.)

**Keywords:** hemoadsorption, heart transplantation, CytoSorb, cardiac surgery, cytokines

## Abstract

Background: Hyperimmune response and cytokine release post-reperfusion might occur after orthotopic heart transplantation (HTx). Intraoperative hemoadsorption (HA) has been introduced to remove such elevated cytokines. We aimed to analyze the effect of intraoperative HA in patients undergoing orthotopic HTx. Methods: Between 2018 and 2022, 40 consecutive orthotopic HTx patients who underwent intraoperative hemoadsorption HA integrated into the cardiopulmonary bypass were compared to 41 historical controls. Primary outcome measures included postoperative hemodynamic stability and blood product requirements, while secondary outcomes were the incidence of acute kidney injury requiring dialysis (AKI-d) and 30-day mortality. Results: Postoperatively, the vasoactive-inotropic score (VIS) did not significantly differ between the groups. However, the use duration for milrinone and dobutamine was shortened by one day compared to controls. The HA group had fewer red blood cell transfusions (765 vs. 1330 mL, *p* = 0.01) and lower fresh frozen plasma requirements (945 vs. 1200 mL, *p* = 0.04). Mechanical ventilation duration was reduced (22 vs. 28 h, *p* = 0.02). AKI-d rates were similar, and 30-day mortality favored non-significantly the HA group (5% vs. 14.6%, *p* = ns). No device-related adverse events were observed. Conclusion: These findings suggest that intraoperative HA might improve immediate postoperative outcomes; however, further validation in larger randomized controlled trials is warranted.

## 1. Introduction

Heart transplantation plays a central role in treating patients with advanced heart failure when other therapeutic options are unavailable or ineffective. Post-transplantation one-year survival rates reach about 90%, with a median survival of 12.5 years [1]. This procedure significantly improves quality of life and functional status, allowing many patients to return to active lives. According to the 2018 International Society for Heart and Lung Transplantation (ISHLT) 35th Adult Heart Transplantation Report, early deaths following heart transplants are predominantly due to graft failure, infections, and multiorgan failure (MOF). Postoperative complications, such as hemodynamic instability requiring high vasoactive/inotropic support, renal dysfunction/failure, and bleeding, can lead to MOF, resulting in mortality rates of up to 20% within the first 30 days post-transplant [2]. Additionally, the limited availability of transplantable donor organs presents a significant obstacle, prompting medical communities to also expand donor criteria, including (among others) increasing the upper age limit for donors [1]. Previous studies have indicated that hemoadsorption during cardiac surgery offers several benefits by moderating the body’s hyperimmune response triggered by major surgical stress and extracorporeal circulation [3,4]. This hyperimmune response causes complement activation and excessive production of inflammatory cytokines due to blood contact with air, foreign materials, and ischemic reperfusion oxidative stress [5,6]. Intraoperative hemoadsorption (HA) in high-risk cardiac surgery patients has been associated with hemodynamic stabilization during the perioperative period, and in line with the reduced need for blood products [7,8,9,10], the later also described in patients operated on under active antithrombotic therapy [11,12].

## 2. Materials and Methods

### 2.1. Patient Selection and Study Design

A retrospective cohort study was conducted using data from departmental databases at the Department of Cardiac and Transplant Surgery, Dubrava University Hospital in Zagreb, Croatia. This study included consecutive patients who underwent orthotopic heart transplantation (HTx) surgery between 15 March 2015 and 8 June 2022, with no major changes in operative protocols over this period. A total of 81 patients were divided into two groups. The control group consisted of 41 patients who underwent orthotopic HTx without the intraoperative use of the hemoadsorber, operated between 15 March 2015 and 31 December 2017. The hemoadsorption (HA) group comprised 40 patients who underwent orthotopic HTx with the CytoSorb^®^ hemoadsorber, operated between 27 January 2018 and 8 June 2022. In terms of induction therapy, all patients received 1 g of mycophenolate mofetil (MMF) preoperatively. After weaning from the cardiopulmonary bypass, 500 mg of methylprednisolone was administered. In the first postoperative days, corticosteroids and thymoglobulin were continued. Subsequently, corticosteroids (prednisolone) were maintained, and the dose was gradually reduced, while cyclosporine was introduced. Thymoglobulin was discontinued once the desired cyclosporine concentration in the blood was achieved.

### 2.2. Ethics Statement

This study is approved by the Ethics Committee of Dubrava University Hospital under the IRB number 2024/1022-17.

### 2.3. Device Information

**Figure 1:**
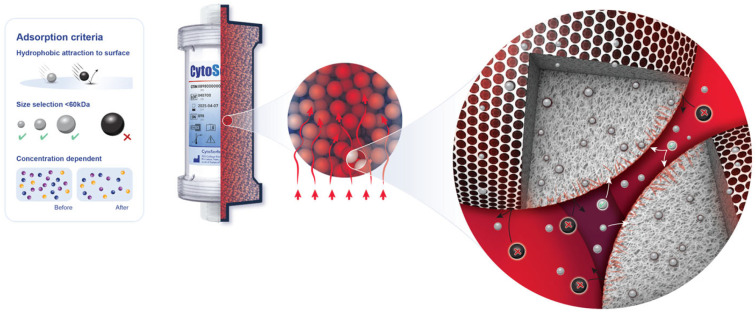
Adsorption properties of polymer beads in CytoSorb^®^.

**Figure 2:**
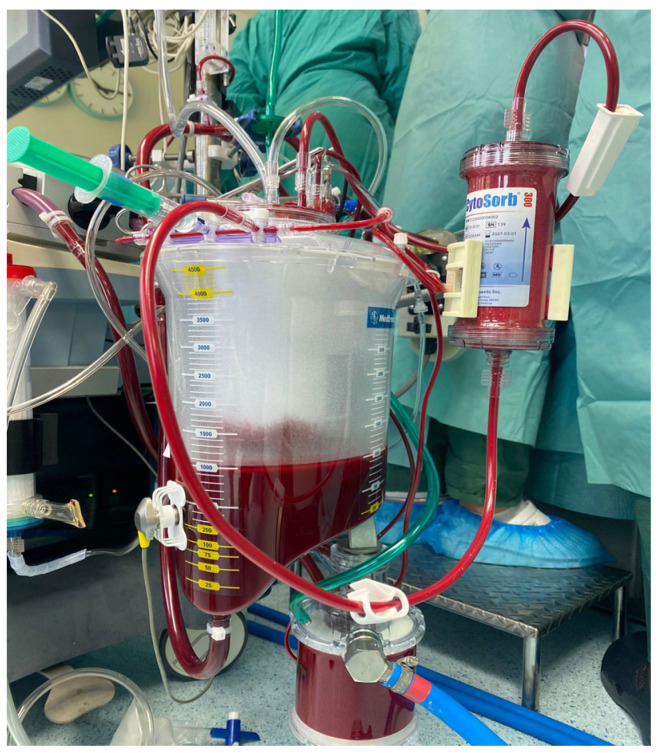
Adjunctive CytoSorb^®^ hemoadsorption device installed into the CPB circuit.

The CytoSorb^®^ device (CytoSorbents, Princeton, NJ, USA) is a cartridge filled with numerous biocompatible porous polymer beads. These beads selectively capture hydrophobic molecules of up to 60 kDa of molecular weight inside their pores (Figure 1). The CytoSorb^®^ column is connected in parallel to the cardiopulmonary bypass (CPB) circuit (Figure 2). The inflow cannula, which directs blood into the device, is positioned downstream from the main pump, while the blood exiting the device returns to the CPB reservoir. As per the manufacturer’s instructions [13], the blood flow through the adsorber should be maintained between 100 and 700 mL/min, which in our procedures typically ranged from 300 to 500 mL/min.

### 2.4. Outcome Measures

The primary outcome was hemodynamic stability in the early postoperative period, reflected by the need for and total duration of vasoactive and inotropic support and the need for blood derivatives.

The secondary outcome measures were the incidence of acute kidney injury requiring hemodialysis and 30-day mortality.

### 2.5. Statistical Analysis

Statistical analysis was performed using the IBM SPSS Statistics software (version 23.0.0.0 for MS Windows, IBM Corporation, Armonk, NY, USA). Fisher’s exact test was used for comparing categorical variables, while the Mann–Whitney U test was applied for continuous variables. Categorical variables are presented as observed frequencies (N) and percentages of the total number of observed events. Continuous variables are presented as medians with interquartile range (IQR) or means with the standard deviation (SD), depending on the normality of the data distribution. In the statistical analyses, a *p*-value of <0.05 was considered statistically significant. A regression analysis was performed to adjust for the potential confounders and assess the independent effect of CytoSorb on clinical outcomes.

## 3. Results

### 3.1. Patient Cohort and Operative Details

In this study, 81 patients have been included, with 41 patients (50.6%) in the control group and 40 patients (49.4%) in the CytoSorb-treated group (HA group). Demographic data, medical history, and operative characteristics are summarized in Table 1.

No significant differences were observed in donor age, body mass index (BMI), reoperations, presence of mechanical circulatory support preoperatively, or duration of cardiopulmonary bypass time. Preoperative laboratory values were largely comparable, except for creatinine, which was significantly higher in the control group (Table S1).

However, the HA group had a significantly higher percentage of females, 14 (35%) vs. 6 (14.6%), and experienced a significantly longer donor heart ischemia time, averaging 47 min more than the control group.

### 3.2. Hemodynamic Stability

Hemodynamic stability in the first six hours postoperatively was assessed with the average doses of inotropic and vasoactive drugs used and the total duration of therapy.

**Figure 3:**
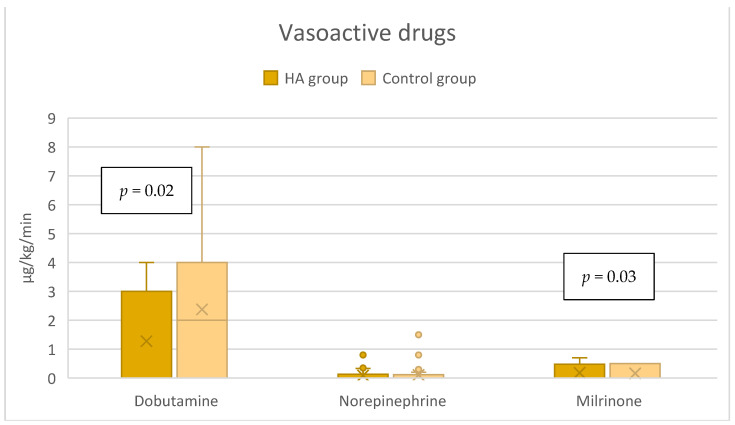
Vasoactive support in the immediate postoperative period (within the first 6 h).

**Figure 4:**
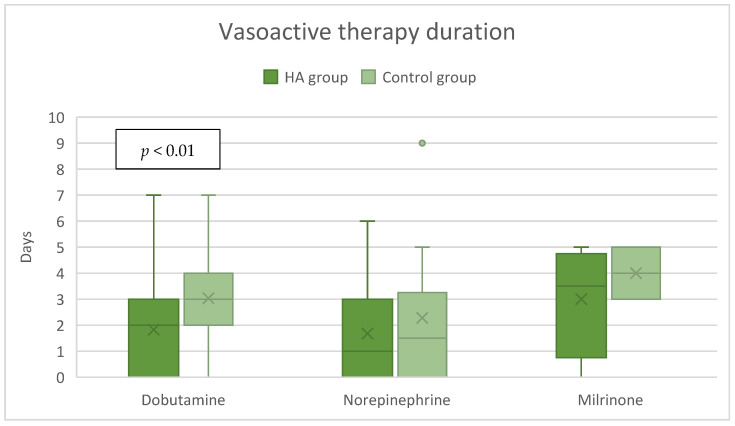
Postoperative vasoactive therapy duration.

In the HA group, the median dose of dobutamine of 0 [0–3] µg/kg/min was significantly lower compared to the control group’s 2 [0–4] µg/kg/min, *p* = 0.02 (Figure 3), while the dose of milrinone was slightly higher, 0 [0–0.5] µg/kg/min vs. 0 [0–0] µg/kg/min, *p* = 0.03 (Figure 3). The duration of both dobutamine and milrinone support was reduced by one day (from 3 to 2 days) in the HA group (Figure 4). A significant difference in norepinephrine dosages between groups was not seen. Still, the therapy duration was half a day shorter with CytoSorb^®^ (1 vs. 1.5 days). Postoperative lactate levels were comparable between groups (Table 2). Additionally, the VIS (vasoactive inotropic score) did not differ between the groups (Table 3).

### 3.3. Blood Product Requirements

**Figure 5:**
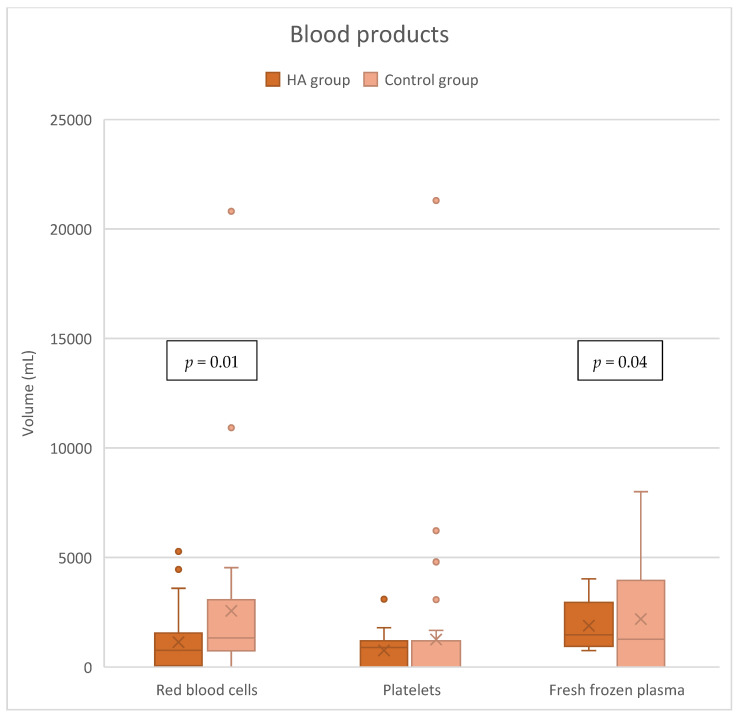
Blood product consumption.

The HA group needed significantly less packed red blood cells (pRBCs) with a median volume of 765 [125–1540] mL compared to 1330 [750–2945] mL in the control group, *p* = 0.01. Also, the need for fresh frozen plasma (FFP) was significantly lower in the HA group, 945 [0–1695] mL vs. 1200 [375–2000] mL, *p* = 0.04. There was no significant difference in platelet consumption between the following groups: 900 [0–1200] mL in the HA group vs. 0 [0–970] mL in the control group, *p* = 0.22 (Figure 5). Preoperative hemoglobin levels were comparable (Table S1).

### 3.4. Acute Kidney Injury Requiring Hemodialysis

The incidence of acute kidney injury (AKI) requiring hemodialysis did not significantly differ between the groups, reporting 8 (20%) in the HA group and 10 (24.4%) in the control group, *p* = 0.8 (Table 3).

### 3.5. Mortality and Other Postoperative Clinical Outcomes

**Figure 6:**
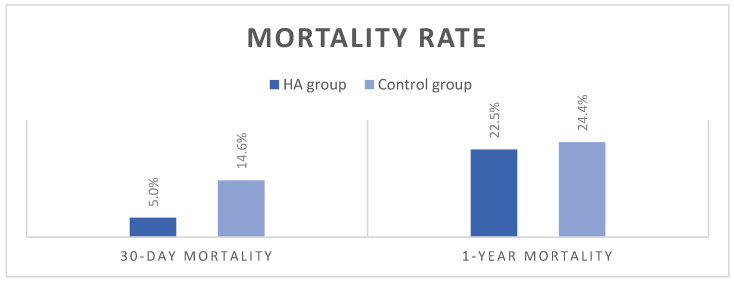
Mortality rates.

Postoperative outcomes are summarized in Table 3. There were no statistically significant differences between groups in the highest postoperative values of inflammatory parameters or the lowest postoperative hemoglobin values (Table S1). The duration of postoperative mechanical ventilation (MV) was significantly shorter in the HA group—22 h compared to 28 h in the control group (*p* = 0.02). As for the intensive care unit and hospital lengths of stay, no significant differences between the groups were observed. The HA group had an increased incidence of rejection on the first biopsy; however, there were no statistically significant differences in rejection rates between groups on the second biopsy. The HA group recorded a lower 30-day mortality rate of 5%, compared to 14.6% in the control group, but the difference did not reach statistical significance (*p* = 0.3). The dominant cause of death was MOF, with no low cardiac output syndrome, deep sternal wound infections, or signs of mesenteric ischemia observed. At 1 year, mortality leveled off between the groups, 22.5% in the HA group vs. 24.4% in the control group, *p* = 1.00 (Figure 6).

There were no adverse events observed attributable to HA.

### 3.6. Regression Analysis (Table S2)

A regression model was performed to evaluate the effect of sex and donor heart ischemic time on primary and secondary clinical outcomes.

Out of patients who received postoperative vasoactive therapy, a linear regression model showed that HA treatment significantly reduced the duration of dobutamine therapy by 1.33 days (*p* = 0.01), but only numerically norepinephrine (*p* = 0.32) and milrinone (*p* = 0.24) by 0.68 and 0.86 days, respectively, compared to controls. Sex and heart ischemic time had no significant effect on the vasoactive therapy duration, although longer ischemia time tended to lead to longer duration of milrinone therapy.

Each 1 h of heart ischemic time significantly increased the average dose of dobutamine use by 0.47 µg/kg/min (*p* = 0.04). After the adjustment of heart ischemic time and sex, HA treatment still demonstrated a significant reduction in the dose of dobutamine used within the first 6 h of HTx (reduction = 1.35 µg/kg/min, *p* < 0.01) but also a significant increase in the dose of milrinone (increase = 0.15, *p* = 0.01).

Multivariate analysis of the effect of treatment and risk factors on the volume of transfusion indicated that the HA treatment significantly reduced the volume of pRBC and/or FFP transfusion. Sex seems to have minimal effect on either pRBC, FFP, or platelet transfusion. The patients with longer ischemic time tended to receive a higher volume of pRBC and FFP transfusions.

After the adjustment for sex and heart ischemia time in the logistic regression model, HA treatment showed a significant reduction in the incidence of postoperative AKI by 67% (OR = 0.33, *p* = 0.03). However, HA treatment showed only a numerical reduction in severe AKI that required dialysis (OR = 0.69, *p* = 0.52).

The models showed that the use of the HA device numerically reduced the 30-day mortality by 69% (OR = 0.31, *p* = 0.19) after adjusting for sex and heart ischemic time. The female patients tended to have a lower 30-day and 1-year mortality rate compared to male counterparts, without statistical significance. Similarly, the longer heart ischemic time increased the 30-day mortality rate by 20% and the 1-year mortality rate by 16%, but none of the increases were statistically significant.

The chance to be positive for the signs of acute cellular rejection during the second biopsy is numerically higher in the HA group than the control group (OR = 2.54, *p* = 0.31) after the adjustment of sex and heart ischemic time. Sex had no contribution to the positive biopsy. Since there was not a single positive result from the first biopsy in the control arm, no logistic model could be performed.

## 4. Discussion

Ischemia-reperfusion injury (IRI) is an inevitable companion of heart transplantation. It encompasses a complex, coordinated interplay of cellular events that trigger the release of cytokines and damage-associated molecular patterns (DAMPs), contributing to systemic inflammation, endothelial dysfunction, and myocardial injury. These pathological changes may lead to acute graft rejection and cardiac allograft vasculopathy [14]. Intraoperative hemoadsorption (HA) with CytoSorb^®^ has been explored as a potential therapeutic approach to mitigate complications associated with IRI in cardiac surgery and transplantation [15,16].

The CytoSorb^®^ device captures hydrophobic molecules of up to 60 kDa (Figure 1), eliminates excessive levels of pro- and anti-inflammatory cytokines (IL-6, IL-10, TNFα, etc.) [17,18], direct-acting oral anticoagulants (DOACs) rivaroxaban, apixaban, edoxaban, and dabigatran, and platelet aggregation inhibitor ticagrelor [19], as well as various toxins and metabolic by-products like myoglobin [20] and bilirubin [21], by surface adsorption and size exclusion [22]. It has also been used intraoperatively during orthotopic HTx surgery with an aim to reduce systemic inflammation and associated complications during the critical perioperative period, potentially improving patient outcomes and survival rates [23]. CytoSorb^®^ comprises highly biocompatible polystyrene-divinylbenzene copolymer porous beads coated with polyvinyl-pyrrolidone, which provide a large surface area (~40,000 m^2^ per cartridge) for adsorption located in the pores’ channels [22]. In cardiac surgery, it is predominantly used intraoperatively, installed in a bypass circuit, providing hemoadsorption of undesirable molecules within the duration of CPB [3].

The use of hemoadsorption in organ transplants has been controversial due to the potential for unwanted drug removal, specifically immunosuppressants. However, a detailed investigation in a large animal model reassuringly reported a minimal level of removal with frequently used immunosuppressant regimens [24]. This group did not observe a significant clearance of prednisolone and basiliximab by CytoSorb^®^, while it accounted for the removal of less than 5% of the daily administered dosages of tacrolimus, cyclosporin A, mycophenolate mofetil (MMF), everolimus, and methylprednisolone. This was also confirmed in an RCT [23] that demonstrated that CytoSorb^®^ did not affect levels of mycophenolic acid, an active metabolite of MMF, often used to prevent organ transplant rejection. There was also no increase in the frequency of early cardiac allograft rejection in the intervention group.

The use of intraoperative hemoadsorption (HA) during orthotopic HTx in our institution has shown promising results in improving patient outcomes, particularly in terms of faster achievement of hemodynamic stability and the decreased need for transfusions. This study aimed to evaluate the impact of HA on these critical factors, which are essential for improving post-transplant recovery and reducing complications associated with heart transplantation.

### 4.1. Hemodynamic Stability

One of the key findings in this study is the reduced length of vasoactive support and the need for dobutamine, associated with HA. Improved hemodynamics are particularly relevant in HTx due to the delicate balance required in managing the newly transplanted heart. Excessive use of vasopressors and inotropes to achieve hemodynamic stabilization, with restrictive fluid administration, inadvertently leads to peripheral hypoperfusion, potentially resulting in acute kidney injury and increased mortality [25,26,27,28].

Vasoplegic syndrome (VS) after HTx is a significant challenge, often arising from the massive release of pro-inflammatory cytokines, ischemia-reperfusion injury, and the physiological stress associated with CPB [29]. In a recent randomized controlled trial (RCT), Nemeth et al. [23] showed that intraoperative HA use during orthotopic HTx was related to a 6.4-fold decrease in the odds ratio of developing VS. In addition, the CytoSorb group had a significantly lower vasoactive-inotropic score (VIS), procalcitonin (PCT) levels, shorter duration of MV, and ICU length of stay. In our study, VIS did not significantly differ between the groups, and the observed differences in doses of the vasoactive medications, particularly milrinone, may have been confounded by potential changes in the vasoactive support practice over time. However, it seems that HA attenuated the effects of excessive release of inflammatory mediators and potentially prevented profound hemodynamic instability, resulting in faster weaning from vasoactive support. Such an outcome is in line with the findings of the abovementioned RCT, even though our cohort included a much sicker patient population with half of the patients being in high urgency status, more than one-third were inotrope-dependent and progressively worsening (INTERMACS class ≤ 3), and a few required mechanical circulatory support preoperatively. Furthermore, as VS is found to be common after HTx [30] and patients who developed VS are more likely to require postoperative ECMO support, renal replacement therapy, reoperation for bleeding, longer mechanical ventilation, and a greater 30-day and 1-year mortality [31], hemoadsorptive treatment may be considered in such patients on a stand-alone platform in the ICU, with careful deliberation on the necessary anticoagulation strategy.

### 4.2. Secondary and Other Outcomes

By stabilizing hemodynamics without the need for excessive pharmacologic intervention and mitigation of the dysregulated inflammation, HA should have contributed to better graft function and early postoperative outcomes. Paradoxically, the incidence of rejection on the first biopsy was more frequent in the HA group without having a clinical impact. This could be due to several factors, including observed preoperative imbalances between the groups (e.g., 47 min longer heart ischemia time in the HA group) and a statistical anomaly of a small sample. In our practice, the first biopsy is performed within two weeks after the transplantation, and the second one within 30 days. There were no statistically significant differences in rejection rates between groups on the second biopsy, and mortality rates were actually numerically in favor of hemoadsorption. In addition, studies that investigated the effect of ischemia time on the graft rejection rate and survival demonstrated a significant increase in adverse outcomes with longer ischemic times [14,32,33]. Furthermore, in patients with myocardial injury, the role of inflammatory mediators in adverse myocardial remodeling and postoperative complications was shown, suggesting that hemoadsorptive interventions on pro-inflammatory mediators might improve postoperative outcomes [34,35].

Moreover, some additional signals of the positive effects potentially attributable to HA were observed. Lactate levels were comparable between the groups despite a significantly longer ischemia time in the HA group. The overall MV duration time was also decreased. The 30-day mortality was almost three times higher in controls. Despite the lack of statistical significance, this finding suggests a potentially clinically relevant trend.

### 4.3. Blood Product Consumption

Another significant finding in this study was the reduced need for blood transfusions in patients who received intraoperative HA. Blood transfusions are often necessary in HTx due to the extensive surgical trauma, bleeding, and coagulation abnormalities that occur during CPB. However, transfusions carry risks such as transfusion-related acute lung injury (TRALI) and increased infection risk that can complicate post-transplant recovery, as well as reduced long-term survival [36,37,38].

The ability of CytoSorb^®^ to reduce the need for transfusions may be linked to its potential to modulate coagulation pathways. By reducing the systemic inflammatory response, HA may help preserve endothelial function and prevent the coagulopathy often seen in heart failure patients [39]. Furthermore, the reduced need for blood products could decrease the risk of transfusion-related complications and improve overall patient outcomes.

The decreased transfusion requirement observed in this study aligns with findings from other studies in which HA has been associated with reduced transfusion rates in cardiac surgery [7,9,40]. While the exact mechanisms are still under investigation, it is possible that the removal of inflammatory mediators and reduction in complement activation play key roles in stabilizing the coagulation system during surgery.

### 4.4. Clinical Implications and Future Directions

The findings of our study are largely aligned with the dominant results of intraoperative HA use in cardiac surgery, particularly in orthotopic HTx. Improved hemodynamic stability and reduced transfusion requirements may lead to shorter ICU stays, faster recovery times, and potentially lower morbidity and mortality rates in orthotopic HTx patients. The beneficial role and safety of HA in HTx are therefore reinforced.

Heart transplantation remains the definitive treatment of advanced heart failure, and survival and quality of life following HTx are excellent [41]. The use of extended donor criteria, procurement of hearts in donation after circulatory death, and transplantation with the use of novel organ preservation techniques will possibly increase the number of organs available. Considering the ongoing unmet need for organs for transplantation, ex vivo organ perfusion with adjunctive hemoadsorptive treatment may play an essential role in combatting organ shortage and early graft rejection [42]. Globally, about half of the HTx recipients are on mechanical circulatory support, usually a left ventricular device, by the time of HTx [43], which augments the risk of bleeding, renal failure, and graft failure in the immediate postoperative period. Adjunctive hemoadsorption may help mitigate these risks, especially in ECMO patients in whom it is associated with accelerated recovery of multiorgan and microcirculatory dysfunction, mitigated inflammatory response, and fewer bleeding complications [44].

### 4.5. Limitations

The main limitations of our study are its retrospective nature with historical controls and the long observational period. These factors inherently limit the ability to draw definitive causal inferences, as several confounding factors that were not controlled for, including changes in clinical practice, patient management, or surgical techniques over time, may have influenced the observed outcomes. It is possible that improvements in standard care over the study period could have contributed to some of the observed benefits in the HA group, independent of the hemoadsorption intervention. Ultimately, the sample size may limit the study’s power to detect subtle differences between groups, particularly in outcomes such as mortality and rejection rates, where statistical significance was not always achieved, despite apparent trends. Nevertheless, this is the largest study in the field of HTx with intraoperative hemoadsorption so far.

Another limitation concerns the baseline imbalances between the groups, particularly the significantly longer organ ischemia time and the higher proportion of female patients in the HA group. Prolonged donor heart ischemia time is a well-established contributor to poorer outcomes [33] and may have influenced some of the conflicting findings presented in this study. A regression analysis suggests that the longer ischemic time in the HA group affected the rejection rate and revealed that intraoperative hemoadsorption was associated with the reduced incidence of postoperative AKI. For the rest of the endpoints, it mostly strengthened the results from unadjusted analyses.

## 5. Conclusions

Intraoperative HA with CytoSorb^®^ appears to offer significant benefits in improving hemodynamic stability and reducing the need for transfusions in orthotopic heart transplant patients. However, while the results are encouraging, prospective studies with larger patient populations are needed to confirm these findings and better understand the mechanisms by which HA improves outcomes. Additionally, the cost-effectiveness of using CytoSorb^®^ should be explored, given the potential benefits of reducing the need for vasoactive therapy and blood transfusions. HA has the potential to become a valuable tool in the management of orthotopic HTx, improving patient outcomes and postoperative recovery.

## Figures and Tables

**Table 1:** Preoperative patient characteristics and operative details.

Variables	HA Group	Control Group	*p*-Value
Patients, n (%)	40 (49.4)	41 (50.6)	1.00
Sex—female, n (%)	14 (35.0)	6 (14.6)	0.04
Age, years	58 [49–62]	57 [48–61]	0.67
BMI, kg/m^2^	26 [23–30]	27 [23–30]	0.87
Patients on high urgency list, n (%)	20 (50.0)	21 (51.2)	1.00
INTERMACS, n (%) 1 2 3 4 5 6 7	0 (0) 2 (5.4) 11 (29.7) 3 (8.1) 2 (5.4) 7 (18.9) 12 (32.4)	n/a 3 (10.0) 9 (30.0) 1 (3.3) n/a 7 (23.3) 10 (33.3)	0.83
Patients requiring MCS, n (%) LVAD ECMO	3 (7.5) 2 (5) 1 (2.5)	1 (2.4) 1 (2.4) 0 (0)	0.36
NYHA class, n (%) I II III IV	0 (0) 3 (7.5) 19 (47.5) 18 (45.0)	0 (0) 10 (24.4) 18 (43.9) 13 (31.7)	0.11
Reoperation, n (%)	8 (20.0)	13 (31.7)	0.31
Donor age, years	46.5 [34–50]	47 [41–55]	0.08
Heart ischemia time, min	174 [107–207]	127 [81–169]	0.01
Surgery time, min	250 [222–300]	295 [250–360]	0.01
CPB time, min	130 [118–148]	130.5 [113–171]	0.73
VIS (intraoperative)	7.25 [4–15]	5 [3–10]	0.28

Legend: HA—hemoadsorption, BMI—body mass index, INTERMACS—Interagency Registry for Mechanically Assisted Circulatory Support, MCS—mechanical circulatory support, LVAD—left ventricular assist device, ECMO—extracorporeal membrane oxygenation, NYHA—New York Heart Association, CPB—cardiopulmonary bypass, VIS—vasoactive inotropic score (VIS = (dobutamine) + (norepinephrine × 100) + (milrinone × 10) + (vasopressin × 10,000) + (epinephrine × 100) + (dopamine)).

**Table 2:** Postoperative lactate levels.

		0 h	6 h	12 h	24 h
Lactate (mmol/L)	HA group	1.9 [1.3–3.9]	4.1 [3.0–5.9]	3.1 [2.4–4.6]	1.9 [1.6–2.5]
	Control group	2.1 [1.5–3.7]	3.09 [2.2–5.9]	3.08 [2.5–3.8]	1.84 [1.4–2.4]
	*p*-value	0.6	0.2	0.8	0.4

**Table 3:** Postoperative clinical outcomes.

Variables	HA Group	Control Group	*p*-Value
AKI incidence, n (%) Requiring hemodialysis, n (%)	12 (30.0) 8 (20.0)	21 (51.2) 10 (24.4)	0.68 0.80
VIS (at 6-h post-op)	5 [3–19]	5 [3–14]	0.75
First biopsy, n (%) ISHLT grade 0 ISHLT grade 1R ISHLT grade 2R	24 (68.6) 8 (22.8) 3 (8.6)	34 (100.0) - -	<0.001
Second biopsy, n (%) ISHLT grade 0 ISHLT grade 1R	25 (80.6) 6 (19.4)	29 (93.5) 2 (6.5)	0.26
Duration of mechanical ventilation, h	22 [14–27]	28 [19–38]	0.01
Length of stay, days ICU Hospital	5 [4–6] 24 [18–26]	5 [4–6] 22 [19–25]	0.80 0.67

Legend: HA—hemoadsorption, AKI—acute kidney injury, ISHLT—International Society for Heart and Lung Transplantation, ICU—intensive care unit, VIS—vasoactive inotropic score (VIS = (dobutamine) + (norepinephrine × 100) + (milrinone × 10) + (vasopressin × 10,000) + (epinephrine × 100) + (dopamine)).

## Data Availability

Data are available on demand due to privacy protection.

## Supplementary Materials

The following supporting information can be downloaded at: https://www.mdpi.com/article/10.3390/jcdd12040119/s1, Table S1: Preoperative and postoperative laboratory parameters; Table S2: Multivariate analysis of the effect of treatment and risk factors on the doses of vasoactive drugs (within the first 6 h)—Linear Regression Results; Table S3: Multivariate analysis of the effect of treatment and risk factors on the vasoactive therapy duration (days)—Linear Regression Results; Table S4: Multivariate analysis of the effect of treatment and risk factors on the volume of transfusion—Linear Regression Results; Table S5: Univariate analysis of mortality rates among female and male patients; Table S6: Multivariate analysis of the effect of treatment and risk factors on 30-day and 1-year mortality—Logistic Regression Results; Table S7: Multivariate analysis of the effect of treatment and risk factors on post-op AKI and AKI requiring hemodialysis—Logistic Regression Results; Table S8: Multivariate analysis of the effect of treatment and risk factors on first and second biopsy positive results—Logistic Regression Results.
