# Supplementary material for: Intraoperative Hemoadsorption in Heart Transplant Surgery: A 5-Year Experience
Source: J Cardiovasc Dev Dis. 2025 Mar 28;12(4):119. doi: 10.3390/jcdd12040119 (PMC12028209; doi:10.3390/jcdd12040119)
Supplement: Supplementary file 1 [file jcdd-12-00119-s001.zip › jcdd-3373856-supplementary.pdf]

Supplement 1. Preoperative and postoperative laboratory parameters

Table S1: Preoperative and postoperative laboratory parameters

Variables	HA Group	Controls	*p*-Value
**Preoperative**			
Creatinine, μmol/L	95.5 [81–117]	114.0 [97–130]	0.01
WBC count, x10⁹/L	7.4 [6–9]	7.8 [7–9]	0.30
CRP, mg/L	5.0 [2–12]	5.0 [2–13]	0.90
Hemoglobin, g/L	139.5 [112–160]	130.0 [113–141]	0.20
**Postoperative**			
Highest postoperative creatinine, μmol/L	134 [116–213]	181 [139–220]	0.07
Highest postoperative WBC count, x10⁹/L	19 [14–24]	16.9 [14–21]	0.42
Highest postoperative CRP, mg/L	136 [88–219]	110 [77–157]	0.18
Lowest postoperative hemoglobin, g/L	86 [77–92]	83 [76–89]	0.42

Legend: HA—hemoadsorption, WBC—white blood cell, CRP—C-reactive protein.

Supplement 2. Regression Analysis: Intraoperative Hemoadsorption in Heart Transplant Surgery:

A 5-Year Experience

1) Sex and Heart Ischemic Time effect on hemodynamics:
   i) VIS score at 0-6 hours after HTx—data severely skewed. The Shapiro–Wilk test for normality is $p<0.0001$ for the original scale and $p=0.0003$ for the log-transformation scale of VIS. A linear regression is inappropriate for this highly skewed data.
   ii) Doses of vasoactive drugs used within the first 6 hours post-op

      (a) Dobutamine:

      Each hour of heart ischemic time significantly increased the average dose of dobutamine use by 0.47 μg/kg/min ($p=0.039$). After the adjustment of heart ischemic time and sex, HA treatment still demonstrated a significant reduction in the dose of dobutamine used within the first 6 hours of HTx (reduction=1.35 μg/kg/min, $p=0.003$).

      (b) Norepinephrine:

      There are only a total of 35 patients with the use of norepinephrine within the first 6 hours of HTx. Longer heart ischemic time tended to increase the dosage use. However, due to the smaller sample size, no statistical significance is shown. HA treatment had no impact on the dose of norepinephrine use.

      (c) Milrinone:

      Even fewer patients received milrinone ($n=28$). The model showed a significant increase in the dose of milrinone after the adjustment of sex and heart ischemic time.

Table S2. Multivariate analysis of the effect of treatment and risk factors on the doses of vasoactive drugs (within the first 6 hours)—Linear Regression Results.

HA Treatment and Risk factors	Parameter Est. (95%CI); p-value **Dobutamine Dose**	Parameter Est. (95%CI); p-value ***Norepinephrine Dose***	Parameter Est. (95%CI); p-value **Milrinone Dose**
**HA vs. Control**	**-1.35 (-2.24, -0.46); p=0.003**	**-0.035 (-0.14, 0.07) ; p=0.501**	**0.15 (0.034 0.27); p=0.012**
Female vs. Male	-0.13 (-1.13, 0.87); p=0.802	-0.018 (-0.13, 0.10) ; p=0.754	-0.01 (-0.14, 0.12); p=0.886
Heart Ischemic Time (per 1 hour increase)	0.47 (0.02, 0.91); p=0.039	0.039 (-0.01, 0.09) ; p=0.140	-0.036 (-0.10, 0.023); p=0.228
R-square/ adj. R-Sq	*0.1288/0.0949*	*0.0298/-0.0080*	*0.0831/0.0474*
OR = odds ratio; CI = confidence interval; HTX = heart transplant; HA = hemoadsorption			

iii) <u>Vasoactive therapy duration</u>

The following analyses are limited to the patients who received vasoactive drugs accordingly.

(a) Dobutamine:

Sex and heart ischemic time had no significant effect on dobutamine duration, while HA treatment reduced the dobutamine therapy duration by 1.33 days compared to controls (p=0.013).

(b) Norepinephrine:

Neither sex, heart ischemic time, nor HA treatment had a statistically significant effect on norepinephrine duration, even though the HA treatment could reduce the duration by 0.68 days (p=0.324).

(c) Milrinone:

Patients with longer heart ischemic time tended to have longer durations of milrinone therapy. HA treatment reduced the milrinone therapy duration by 0.86 days, numerically (p=0.235).

Table S3. Multivariate analysis of the effect of treatment and risk factors on the vasoactive therapy duration (days)—Linear Regression Results

HA Treatment and Risk factors	Parameter Est. (95%CI); p-value **Dobutamine duration**	Parameter Est. (95%CI); p-value ***Norepinephrine Duration***	Parameter Est. (95%CI); p-value **Milrinone Duration**
**HA vs. Control**	**-1.33 (-2.37, -0.29); p=0.0134**	**-0.68 (-2.05, 0.69) ; p=0.324**	**-0.86 (-2.32 0.59); p=0.235**
Female vs. Male	0.77 (-0.41, 1.94); p=0.196	0.31 (-1.48, 2.10) ; p=0.725	-1.06 (-2.71, 0.58); p=0.197
Heart Ischemic Time (per 1 hour increase)	0.16 (-0.35, 0.67); p=0.525	0.019 (-0.67, 0.70) ; p=0.957	0.72 (0.05, 1.40); p=0.037
R-square/ adj. R-Sq	*0.1349/0.0809*	*0.0257/-0.0492*	*0.1960/0.1156*

OR = odds ratio; CI = confidence interval; HTX = heart transplant; HA = hemoadsorption

2) Sex and Heart Ischemic Time effect on the volume of transfusion:
   i) pRBC (packed red blood cells):
   The model shows that the treatment and length of heart ischemic time are two significant contributors to the volume of pRBC consumption. HA treatment reduced pRBC consumption by 1795 mL (p=0.007), and 1 additional hour of heart ischemic time could increase the pRBC requirement by 858 mL (p=0.009).
   ii) FFP (fresh frozen plasma):
   A similar finding was observed in FFP transfusion. HA treatment reduced FFP consumption by 989 mL (p=0.026), and 1 additional hour of heart ischemic time could increase the pRBC requirement by 435 mL (p=0.049).
   iii) PLT (platelets):
   Neither sex (p=0.910), heart ischemic time (p=0.224), nor treatment (p=0.254) had a statistically significant effect on platelet transfusion.

Table S4. Multivariate analysis of the effect of treatment and risk factors on the volume of transfusion—Linear Regression Results

HA Treatment and Risk factors	Parameter Est. (95%CI); p-value **RBC volume**	Parameter Est. (95%CI); p-value **FFP volume**	Parameter Est. (95%CI); p-value **Platelet volume**
**HA vs. Control**	**-1795 (-3084, -505); p=0.007**	**-989 (-1858, -121) ; p=0.026**	**-692 (-1892, 508); p=0.254**
Female vs. Male	-289 (-1739, 1162); p=0.693	-549 (-1526, 429) ; p=0.267	-77 (-1427, 1273); p=0.910
Heart Ischemic Time (per 1 hour increase)	858 (216, 1500); p=0.009	435 (2.7, 867) ; p=0.049	367 (-230, 965); p=0.224
R-square/ adj. R-Sq	*0.1356/0.1019*	*0.1059/0.0711*	*0.0286/-0.0093*
OR = odds ratio; CI = confidence interval; HTX = heart transplant; HA = hemoadsorption			

3) Sex and Heart Ischemic Time effect on 30-day/1-year mortality
   a) Even though the 20 females were unevenly distributed in the HA (n=14, 35.0%) and control arms (n=6, 14.6%), a univariate analysis showed that there was no statistically significant difference in terms of 30-day mortality and 1-year mortality among females and males, regardless of treatment assignment.

Table S5. Univariate analysis of mortality rates among female and male patients

Sex	30-day death rate	1-year death rate
Male	11.5% (7/61)	25.0% (15/60)
Female	5% (1/20)	20.0% (4/20)
Fisher's exact chi-sq. test	p-value=0.672	p-value=0.768

b) A logistic regression model was performed to evaluate the effect of sex and heart ischemic time on 30-day and 1-year mortality, separately.
The models show that the use of the HA device numerically reduced the 30-day mortality by 69% (OR=0.31, p=0.193) after adjusting for sex and heart ischemic time. The degree of reduction in 1-year mortality diminished (OR=0.91, p=0.868). The female patients tended to have a lower 30-day and 1-year mortality rate compared to male counterparts, without statistical significance. Similarly, the longer heart ischemic time increased the 30-day mortality rate by 20% and the 1-year mortality rate by 16%. But none of the increases were statistically significant.

Table S6. Multivariate analysis of the effect of treatment and risk factors on 30-day and 1-year mortality—Logistic Regression Results

HA Treatment and Risk factors	OR (95%CI); p-value<br>**30-day Death**	OR (95%CI); p-value<br>**1-year Death**
**HA vs. Control**	**0.31 (0.05, 1.81); p=0.193**	**0.91 (0.30, 2.76) ; p=0.868**
Female vs. Male	0.54 (0.06, 4.93); p=0.583	0.73 (0.20, 2.65) ; p=0.629
Heart Ischemic Time (per 1 hour increase)	1.20 (0.54, 2.65); p=0.651	1.16 (0.67, 2.01) ; p=0.596
c-statistic	*0.664*	*0.549*
OR = odds ratio; CI = confidence interval; HTX = heart transplant; HA = hemoadsorption		

4) Sex and Heart Ischemic Time effect on AKI requiring hemodialysis (AKI-D)

After the adjustment of female and heart ischemic time, HA treatment showed a significant reduction in the incidence of post-op AKI (postoperative acute kidney injury) by 67% (OR=0.33, p=0.033). However, HA treatment only showed a numerical reduction in AKI-D (OR=0.69, p=0.523). This finding remains consistent with the result without the adjustment.

Table S7. Multivariate analysis of the effect of treatment and risk factors on post-op AKI and AKI requiring hemodialysis—Logistic Regression Results

HA Treatment and Risk factors	OR (95%CI); p-value **Post-op AKI**	OR (95%CI); p-value ***AKI requiring hemodialysis***
**HA vs. Control**	**0.33 (0.12, 0.92); p=0.033**	**0.69 (0.22, 2.16) ; p=0.523**
Female vs. Male	0.85 (0.28, 2.59); p=0.780	0.85 (0.23, 3.12) ; p=0.803
Heart Ischemic Time (per 1 hour increase)	1.44 (0.87, 2.36); p=0.154	1.29 (0.74, 2.27) ; p=0.373
c-statistic	*0.657*	*0.549*
OR = odds ratio; CI = confidence interval; HTX = heart transplant; HA = hemoadsorption		

5) Sex and Heart Ischemic Time effect on other outcomes
   i) Mechanical ventilation (MV) duration—data severely skewed. The Shapiro–Wilk test for normality is $p<0.0001$ for both the original scale and log-transformation scale of MV duration. A linear regression is inappropriate for this highly skewed data.
   ii) Heart transplant graft cellular rejection (HTR)
      (a) Since there is NO single positive result from the 1st biopsy in the control arm, no logistic model could be performed.
      (b) The chance to be positive during the second biopsy is numerically higher in the HA group than the control group (OR=2.54, p=0.309), after the adjustment of sex and heart ischemic time. Sex has no contribution to the positive biopsy. The longer ischemic time could cause a numerically higher positive rate.

Table S8. Multivariate analysis of the effect of treatment and risk factors on first and second biopsy positive results—Logistic Regression Results

HA Treatment and Risk factors	OR (95%CI); p-value **first biopsy positive**	OR (95%CI); p-value ***second biopsy positive***
**HA vs. Control**	N/A since there is no positive case (n=0) in the control arm	**2.54 (0.42, 15.28) ; p=0.309**
Female vs. Male		1.00 (0.17, 5.73) ; p=0.998
Heart Ischemic Time (per 1 hour increase)		1.69 (0.71, 3.99) ; p=0.233
c-statistic	*0.657*	*0.711*
OR = odds ratio; CI = confidence interval; HTX = heart transplant; HA = hemoadsorption		
